# The Mental Health of Farmers and Farmworkers Impacted by Flooding and Drought: Protocol for a Mixed Methods Study

**DOI:** 10.2196/73827

**Published:** 2025-12-25

**Authors:** Daniel Mark Blake, Katya Brooks, Jennifer Israelsson, Rhiannon Cordiner, Anusha Rajamani, Sari Kovats, Paul Coleman

**Affiliations:** 1 UK Health Security Agency London United Kingdom; 2 London School of Hygiene & Tropical Medicine London United Kingdom

**Keywords:** mental health, well-being, flooding, drought, water, resilience, climate change, agriculture, farming

## Abstract

**Background:**

Farmers and farmworkers in England are a population group who are particularly vulnerable to the mental health impacts of environmental stressors, including flooding and droughts. While international studies, particularly from Australia, have examined these impacts, there is a critical gap in understanding the mental health consequences of such events for England’s farming community. This gap is particularly concerning given the increase in frequency and severity of flooding and drought events due to climate change, which will have significant repercussions for the mental well-being of those whose livelihoods depend on affected land.

**Objective:**

This study aims to investigate the mental health impacts of flooding and drought events on farmers and farmworkers in England. It will examine how these events affect their livelihoods and explore both compounding and mitigating factors associated with mental health challenges.

**Methods:**

A mixed methods approach will be used, beginning with a national online survey distributed via trusted intermediary agricultural organizations from January 2025 to March 2025. The survey will include the Warwick-Edinburgh Mental Wellbeing Scale to assess participants’ mental well-being. A subset of respondents will be selected for follow-up semistructured interviews in late 2025 to early 2026 to gather more in-depth data. Data will be thematically analyzed, allowing for the identification of key patterns in mental health impacts, coping mechanisms, and resilience-building strategies.

**Results:**

Survey and interview data will be analyzed during 2025 and 2026 to identify themes and patterns related to the mental health challenges faced by the farming community. This will include exploring coping mechanisms, support networks, and resilience-building strategies, with findings used to inform national interventions. Data obtained from the Warwick-Edinburgh Mental Wellbeing Scale component will be assessed against results from existing studies that have used the scale for other population groups to provide comparative results.

**Conclusions:**

Findings from this study will inform the development of health protection interventions, supporting the farming community in building resilience to future flooding and drought events. This study will also provide valuable insights into the mental health impacts of flooding and droughts in England, contributing to the growing international research on the mental health effects of climate change.

**International Registered Report Identifier (IRRID):**

DERR1-10.2196/73827

## Introduction

### Background

Exposure to flooding and drought events is already an increasing issue for health and well-being in the United Kingdom. This trend is expected to continue because of further climate changes, with warmer and wetter winters and hotter and drier summers projected [1]. Using the Representative Concentration Pathway 8.5 high emissions scenario in which the world continues to create high levels of emissions, UK Climate Projections data show that, by 2070, winters in the United Kingdom are projected to be up to 4.5 °C warmer and 30% wetter than they were in 1990. Summers in the United Kingdom may be up to 6 °C warmer and 60% drier [2].

The agricultural sector in England is highly susceptible to the effects of flooding and droughts. Farmers and farmworkers, whose livelihoods depend directly on the land, are particularly vulnerable to these events, which can disrupt agricultural production, damage infrastructure, and lead to financial loss. The resulting strain on physical and economic resources can also have significant consequences for mental health, contributing to stress, anxiety, depression, and other psychological challenges [3,4].

### Flooding Impacts

Coastal, fluvial, and surface water flooding can all lead to “significant” impacts according to the National Risk Register [5], with likelihoods of significant impact currently at approximately 1% to 5% for all 3 flood types over a 5-year period in the United Kingdom. The third UK Climate Change Risk Assessment [6,7] published in 2023 and the National Risk Register [5] have identified flooding as one of the most important climate change adaptation challenges facing the United Kingdom. In all future climate change scenarios, direct and indirect flood risks are projected to rise over the 21st century. Increasingly higher average rainfall and changing rainfall patterns, alongside rising sea levels, will contribute to a projected rise in the frequency and severity of flood events.

While the greatest expected annual damage is likely to remain being caused by river flooding until at least the 2080s, the greatest increase in expected annual damage risk is projected to be from surface water and coastal flooding [7]. The increase in flood frequency and severity will likely lead to a corresponding increase in knock-on health impacts from coinciding threats, such as landslides and critical infrastructure service damage or disruption.

### Drought Impacts

As assessed by the 2025 National Risk Register [5], drought events could have wide-ranging impacts between “minor and catastrophic,” with likelihood levels between <0.2% and 5% (occurrence over 5 years) in the United Kingdom. However, “the future risk of droughts due to climate change is increasing, and there is a trend towards hotter summers with associated increasing water demand. Simultaneously, changes in consumer habits and population growth are increasing water use in the UK” [5]. Droughts, in contrast to flooding, are often slow-onset, complex phenomena determined by a combination of rainfall, evaporation, water demand, and local ground and environmental conditions.

Multiple definitions of drought exist, commonly split into the 4 categories of hydrological, meteorological, agricultural, and ecological drought depending on whether and how impacts are considered [8]. Although there are many indicators available for each category, particularly for meteorological and hydrological droughts, there is still no consistent approach to characterize agricultural and ecological droughts [9].

Similar to flooding, droughts also have the potential for compounding and coinciding hazards, including heat waves and wildfires [10]. However, there are difficulties in identifying impacts and documenting health effects from droughts due to their slow onset and given the complexities in assigning a defined beginning and end to droughts, where effects tend to accumulate over time [11].

### Mental Health of Farmers and Farmworkers

Although some studies suggest lower general rates of depression and anxiety among farmers than among other population groups [12], far more studies and surveys indicate the opposite [13-15]. Lower rates of anxiety and depression in farmers in certain areas on a day-to-day basis may be attributable to the resilience derived from the characteristics of farming families in some communities [14]. In addition to depression and anxiety, other mental health impacts on farmers following flooding and drought may include posttraumatic stress disorder and suicide [16], social isolation [17], stress and worry over loss of income, and penalties for not meeting contractual agreements with supermarkets [9]. Indeed, the mental health impacts of droughts and flooding for farming populations can be particularly intertwined with various socioeconomic circumstances as well as environmental and community factors.

An integrative literature review on the mental health impacts of climate change among vulnerable populations worldwide [18] found that the most impactful and well-documented manifestation of climate change for mental health is drought and lack of water resources. An English national study of flooding and health that surveyed people in neighborhoods affected by flooding in the winters of 2013 to 2014 and 2015 to 2016 revealed that there was an adverse impact on mental health among those whose lives were disrupted by flooding as well as those whose homes were flooded [19]. Farmers and farmworkers are a group who are particularly exposed to health impacts from flood and drought events due to their livelihoods being so closely dependent on the land and agricultural output [20]. A systematic review conducted by Vos et al [21] in 2021 also identified mental health as a major health theme of importance during droughts and farmers as a rural community that is particularly vulnerable to the mental health impacts of drought.

### Knowledge Gap and Rationale

Most of the evidence for mental health impacts to farmers has been focused on drought in Australia. In Australia, drought is commonly defined as hot and dry drought usually associated with summer. However, in the United Kingdom, where reservoir and groundwater levels generally rise during the autumn and winter, a winter (or multiple winters) with low rainfall can lead to slow-onset drought events that have a significant impact [22]. Flooding also occurs in a variety of types in the United Kingdom, with multiple and complex impacts for farmers. Land flooded by sea water in coastal and estuarine environments, for example, often requires many years to recover due to soil damage from salinization. In addition to direct impacts from floodwater inundation, high-velocity fluvial and surface water flooding can cause riverbank and ground surface erosion and the loss of farmland, topsoil, infrastructure, livestock, and crops. Impacts such as those from soil erosion may be exacerbated by repeated flood events or land use practices occurring in some parts of the country.

While there is evidence worldwide to demonstrate the mental health impacts of droughts on at-risk groups, including farmers and farmworkers, there is currently a gap in knowledge assessing the impacts specifically for the United Kingdom [23,24]. The UK Health Security Agency’s Health Effects of Climate Change in the UK report states that “the greatest health impacts of flooding in the UK are on mental health” [25]. However, there remains a particular gap in knowledge when considering the mental health impacts on farmers and farmworkers from flooding in the United Kingdom. While there may be benefits from taking lessons from studies focused overseas and more general studies in the United Kingdom, impacts on health vary between populations and at-risk groups for reasons related to population vulnerability and the types of flooding or drought events [26].

The 2024 farmer confidence survey carried out by the United Kingdom’s National Farmers’ Union (NFU) shows confidence levels at record lows, with extreme weather the leading concern with 82% of participants citing this as having a “very negative” or “fairly negative” impact on their business in the previous year [27]. Further severe flood events occurred in the United Kingdom after the 2024 NFU survey. With both drought and heavy rainfall predicted to become more common and intense in the United Kingdom due to climate change, the NFU is promoting a holistic water management practice, including the management of both too much and too little water together [28]. Due to the numerous routes through which water management issues could impact mental health and well-being, they recommend that flooding and drought impacts should be monitored and researched together. We adopt the combined approach to flooding and drought mental health impacts in this study.

### Aim

This study aims to fill the existing knowledge gap by investigating the mental health impacts of both flooding and drought on farmers and farmworkers in England. Through the exploration of the direct and indirect effects of these events, as well as the factors that contribute to or mitigate mental health challenges, the implementation of the protocol will provide valuable insights for policymakers, health professionals, and agricultural support organizations, as well as farmers and farmworkers themselves.

### Objectives

The objectives of our mixed methods study to assess the impact of flooding and drought on the mental health of farmers and farmworkers in England are to (1) investigate how farmers and farmworkers and their lives and livelihoods are impacted by flooding and droughts, (2) explore the perceived impacts of flooding and droughts on the emotional and social well-being of farmers and farmworkers, (3) investigate the factors that directly or indirectly compound the mental health impacts of flooding and droughts (eg, preexisting physical or mental health conditions and external stressors, including socioeconomic circumstances), and (4) examine the factors that may mitigate the root causes of mental health impacts from flooding and droughts.

## Methods

**Figure 1:**
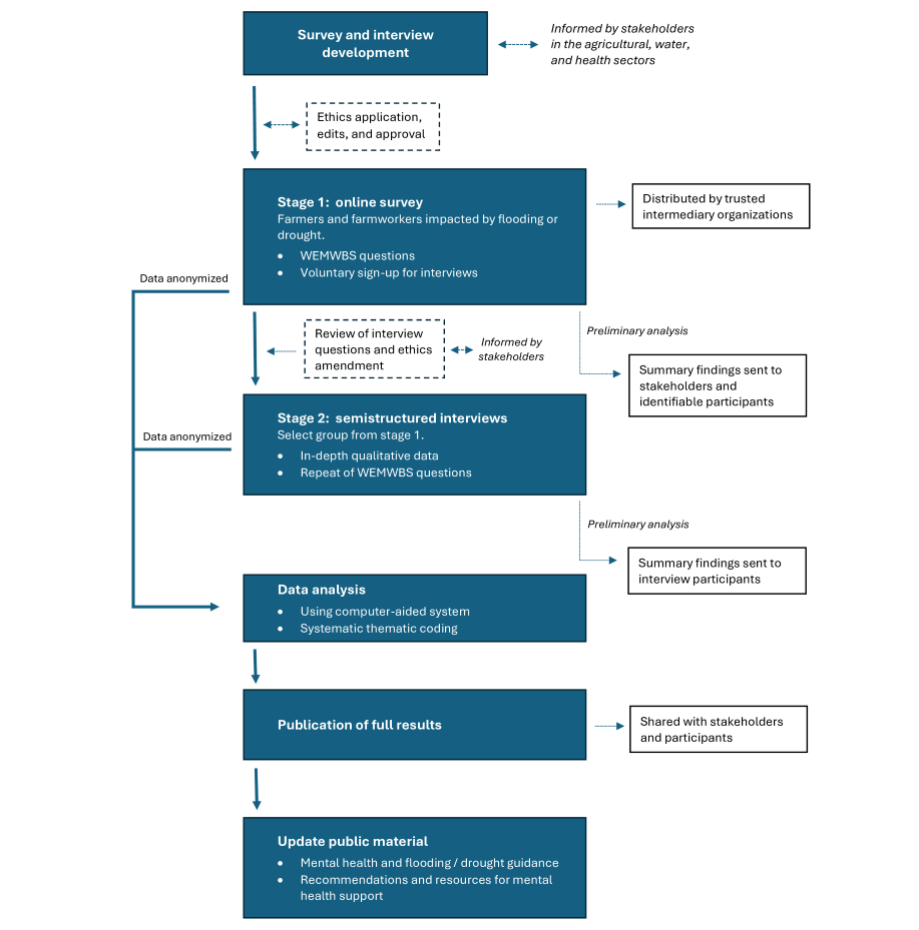
Study phases for assessing the impact of flooding and drought on the mental health of farmers and farmworkers in England. WEMWBS: Warwick-Edinburgh Mental Wellbeing Scale.

This study uses a sequential mixed methods approach. Data collection is broken down into 2 core phases that are shown in Figure 1: an online survey and semistructured interviews.

### Stage 1: Online Survey

The primary method being used for data collection is an online survey compiled through Microsoft Forms and accessed through a shortened Microsoft Forms URL. This method has been identified to have multiple advantages in the population of interest. Surveys allow for a large amount of data to be collected quickly and allow participants to complete them in their own time, with both open- and closed-ended questions to capture quantitative and qualitative data. Responding to surveys is also one of the quickest ways to collect data, which addresses the engagement barrier of being “too busy” identified by Lyon et al [29] in 2020. A postal survey will not be conducted due to limited resources and capacity and because this was deemed unnecessary for the intended audience following discussions with multiple stakeholders in the agricultural sector. The survey is mobile phone–compatible as this was deemed important to maximize the response rate.

Data will be collected at the household level to allow for exploration of factors affecting other members of the family or household. A dedicated contact will be available for those who may have questions about how to complete the survey or other research processes. Survey data will be collected from a questionnaire on the following themes, with individual questions outlined in Multimedia Appendix 1 and guided by feedback from stakeholders in the agricultural, health, and water sectors:

- Demographics and farm information (including farm details)
- Experience with flooding and drought
- Mental health impacts and coping strategies
- Warwick-Edinburgh Mental Wellbeing Scale (WEMWBS)
- Open feedback and option for contribution to the interview component
- Mental health resources and support

The survey will enable a broad overview of the mental health implications for farmers and farmworkers across England, with potential trends identified. The survey will also be used to recruit for follow-up semistructured interviews through a voluntary component of the questionnaire that explains this and allows participants to enter contact details. We recognize that caution is required regarding the timing of surveys or other assessments of mental health related to disasters as they may coincide with other circumstances unrelated to the disasters. Additionally, surveys conducted soon after a disaster (or multiple disasters) may coincide with specific psychological phases of the typical disaster recovery cycle [30] and large variations in corresponding emotional highs and lows.

From the reviewed literature, the most used mental health assessment tool was the 10-question Kessler Psychological Distress Scale [31]. However, as most studies reviewed were conducted in Australia, this study will instead use a tool that has been more commonly used in UK studies, including with farming populations [15], to improve the analysis: the validated 14-item WEMWBS[32]. The WEMWBS comprises only positively worded items related to different aspects of mental health [32]. The WEMWBS was developed by an expert panel drawing on academic literature, qualitative research with focus groups, and psychometric testing of an existing scale. It has been validated on different populations, including university students [33], secondary mental health care users aged ≥18 years [34], the general population [32], and the UK farming population [35]. In its original UK validation study, the Cronbach α scores were 0.89 and 0.91 across student and general population samples, respectively, indicating good interitem homogeneity [32]. Test-retest reliability at 1 week was high (0.83), and the WEMWBS correlated positively with established scales such as the World Health Organization–Five Well-Being Index (*r*=0.77; *P*<.01) [32]. The WEMWBS has been used in several studies that have included subsequent interventions, and Blodgett et al [36] conducted a review to assess the effectiveness of different intervention types. These may usefully inform the development of resilience-building strategies that follow from this study. The survey will allow for the collection of WEMWBS data on mental health experiences within the farming population that can be compared to those other population types in the United Kingdom. It is recognized that these data will be quite broad given the variety of participants who may complete the survey. Nevertheless, they will provide a valuable opportunity to compare with existing WEMWBS datasets.

### Stage 2: Semistructured Interviews

Participants will be invited to a 1-hour semistructured interview via Microsoft Teams, and previous consent will be sought. Interviews will enable the collection of more in-depth qualitative data regarding mental health impacts, coping mechanisms, and other stressors. The WEMWBS will again be used as part of the stage 2 interview questions. This will allow for the collection of WEMWBS data from targeted participants, ensuring a coverage of different farm types and sizes (based on the UK government 2024 farm classification [37]) and further information from respondents about the sources of distress. In addition to comparisons to WEMWBS findings from other published studies, the collection of WEMWBS data from the interviews will allow for comparisons to the initial WEMWBS data collected from the same participants during the stage 1 survey. This will prove useful given the different data collection techniques and the fact that floods or droughts may occur in between stages 1 and 2 of data collection.

Alongside stressors, coping mechanisms will be further explored to better understand what interventions could be most useful. These factors have been informed by previous research, including that by Staniford et al [38] in 2009, Wheeler et al [39] in 2018, and Lyon et al [29] in 2020. The interviews will allow us to use an open framework to engage in guided discussion. Importantly, this format of questions gives space for the interviewee to influence the focus and encourage dialogue with the interviewer [40].

An interview protocol and topic guide based on a schedule of common questions and informed by responses from the stage 1 survey will be piloted and used to ensure consistency and enable comparison across interviews. However, the following are the main criteria, with initial proposed question themes outlined in Multimedia Appendix 2:

- Demographic information—updates following stage 1 and discussion
- Personal mental health impacts and coping mechanisms
- WEMWBS
- Farm impact and resilience measures
- Community (including family) impact and resilience measures
- Resources and assistance
- Policy and advocacy
- Outlook and open discussion

A summary of key findings will be shared with participants after both the survey and interviews.

### Participants

The participant group for both stages of this study is farmers and farmworkers working on multiple farm types and sizes across England [37]. England has been chosen due to the remit of the UK Health Security Agency only covering this country of the United Kingdom.

Inclusion criteria are (1) farmers and farmworkers in England, defined as those who own, live on, or are involved in the day-to-day management of the farm (this includes farm owners and operators, farmworkers, and tenant farmers and contractors); (2) farmers with all farm types (ie, cereals, horticulture and potatoes, specialist pigs, specialist poultry, dairy, upland grazing livestock, lowland grazing livestock, and other; adapted from the UK farm classification [37] through stakeholder engagement); and (3) age of ≥18 years.

Farmers or farmworkers with farms that have been impacted by flooding or drought are being actively encouraged to participate.

Exclusion criteria are (1) casual and seasonal farmworkers (ie, those who do not spend most of their working hours on the farm throughout the year), (2) children under the age of 18 years (data will not be collected directly from children; however, adults of the household may wish to include their views in the survey), (3) those who speak languages other than English due to limitations of the research team and translation capacity, and (4) agriculture types that are not typically classed as a type of farming by the UK government farm classification system [37] (eg, forestry, agroforestry, and aquaculture).

### Participant Recruitment

Previous studies have shown that farmers prefer to be approached by and engage with people or organizations whom they already have a connection to and who have knowledge about farming [41]. Therefore, links and information to the stage 1 survey are being distributed through a variety of communication channels by intermediary organizations already engaged with the community in the farming and agricultural sector. This includes existing newsletters, social media, and internal communication methods through office holders and their staff at both regional and national levels. Intermediary organizations will not have knowledge of who has chosen to complete the survey.

Survey respondents will be invited to participate in a follow-up interview (stage 2) to be conducted within the following 12 months, and a subset of up to 10 respondents will be selected for interview. Participants for the interview stage will be selected at random, although the research team will check that the random selection covers a range of demographics, regions, and farm types. If not, another random selection will be generated.

### Participant Sample

Convenience sampling with snowballing will be used in this study. This allows for quick and easy access to participants of interest and for additional participants to be identified. This method has been chosen due to the population’s expressed preference to work with organizations that they are already involved with and being an underrepresented population for mental health considerations [41].

Recognizing the differences in flooding and drought events experienced across England, a geographical spread of participants from across the country will be sought. Where possible, minimum sample sizes of 300 for the stage 1 survey and 12 for the stage 2 interview will be sought. A sample size of 300 is often considered a “good” sample size for survey research [42,43], and Guest et al [44] found that most (97%) key research themes are captured in 12 interviews.

This survey description and design will naturally target those affected by flooding or drought rather than collecting data that assess mental health more generally within the population. Additionally, the methods used to recruit participants (including through intermediary organizations that focus on providing support to affected individuals and communities in the agricultural sector) will mean that a large proportion of the population surveyed and interviewed will be those already affected by flooding or drought and who have already sought support. This will be acknowledged in the study write-up.

### Data Analysis

The statistics functions on Microsoft Forms will be used for preliminary analysis of stage 1 survey data to identify headline themes that are emerging. Stage 1 data will be exported in the Microsoft Excel format for further analysis.

Stage 1 and 2 datasets will be thematically analyzed and coded using the NVivo (Lumivero) software and a coding framework constructed through an iterative process that will be both inductive and deductive and trialed and agreed to by the research team.

Thematic analysis will identify recurring themes, patterns, and key experiences related to the mental health challenges that farmers face during flooding and droughts. Comparisons will be made across different demographic groups, regions, and types of farming to explore any variations in mental health impacts. This approach will provide a deep understanding of the emotional, psychological, and social effects of environmental stressors on farmers, supporting the overall aims of this study.

### Ethical Considerations

This study involves humans and the surveying and discussion of potentially sensitive topics. Ensuring that this potentially sensitive research is conducted ethically and avoidance of any harmful impact on participants is of paramount concern to the research team. This study adheres to appropriate ethical review and approval as per institutional guidelines. Ethics approval for this study has been granted by the UK Health Security Agency’s Research Ethics and Governance Group (reference R&D601; November 11, 2024).

In recognition that the subject matter and qualitative interview process could be reflective and emotional for interviewees and may elicit sensitive and personal information and “unanticipated themes,” this study has been divided into 2 distinct and optional stages. Participants will be provided with information sheets and consent forms in advance of each stage that will outline and ensure understanding of (1) details of the study, (2) the fact that participation in only the second stage will involve taking part in an interview and that this will involve participating to discuss experiences and views on mental health impacts and will last for up to 1 hour, (3) the fact that participation in this study is entirely voluntary and that they can withdraw from the study up until the point that the data are not identifiable by the research team without giving a reason, and (4) the fact that they are welcome to discuss any concerns about the study or participation with any member of the research team.

Information on mental health support and resources available for participants has been drafted by the research team in collaboration with multiple stakeholders, including mental health and farming-focused organizations, and will be provided at the start and end of both study stages (Multimedia Appendix 3).

Data collected from the survey will be stored in a deidentified format. Regarding the collection of secondary personal identifiable information for the purpose of making contact for interviews, these details will be stored in a separate document and not be added to questionnaire response documents.

Interviews will be recorded and transcribed verbatim for data analysis using the postmeeting transcripts available through Microsoft Teams, with any necessary corrections to wording made by the member of the research team who conducted the interview. All research output materials will be anonymized, and participants will not be identifiable within the research outputs. All data will only be accessible to the members of the research team. Any data published as a part of the analysis in reports or peer-reviewed papers will be anonymized.

## Results

### Stage 1: Online Survey

This survey was distributed through trusted intermediary organizations from January 2025 to March 2025. As of February 12, 2025, a total of 58 participants had completed the survey. Responses so far cover most regions of England, providing a broad overview of the mental health impacts of flooding and drought across the country’s agricultural sector. Data analysis will focus on identifying trends in mental well-being and mental health issues, with particular attention to how different types of flooding and drought events have affected various farming communities. Demographic factors such as age, gender, and farm size will also be considered to explore any potential disparities in mental health outcomes. Results from the WEMWBS questions will be compared to those from other studies that involve both the agricultural sector and other population groups [32,34,35] to compare mental health and well-being outcomes.

### Stage 2: Semistructured Interviews

At least 10 participants will be selected for semistructured interviews to be held in late 2025 to early 2026, with the aim of collecting more detailed qualitative data on the mental health impacts of flooding and drought. As of February 12, 2025, a total of 20 survey respondents had volunteered to participate in further research, including interviews. The interviews will explore the emotional and psychological challenges faced by farmers and farmworkers, as well as the coping mechanisms they have used. The thematic analysis of the interview data alongside the stage 1 survey data will provide a deeper understanding of the specific stressors and support systems that influence mental health in the farming community. Results from the WEMWBS questions in this part of the study will also be presented, allowing for a longitudinal comparison with those from the stage 1 survey, the consideration of any cascading or compounding events in between with potential for long-lasting impacts on mental health [45,46], and comparisons with those of previous studies. Results from both stage 1 and stage 2 are expected to be published in mid- to late 2026.

## Discussion

### Principal Findings

This study will provide critical insights into the mental health impacts of flooding and drought on farmers and farmworkers in England. Through the use of both quantitative and qualitative methods, this study will identify common themes and challenges while also allowing for a nuanced exploration and discussion of individual experiences. The findings are expected to highlight the importance of mental health support services and mechanisms that can be tailored to the needs of farmers and farmworkers in England.

### Comparison With Prior Work

Previous research on the mental health impacts of climate change has primarily focused on droughts in Australia, where the agricultural sector faces different challenges compared to the that in United Kingdom. This study will contribute new data specific to the UK context, where farmers face a more diverse range of climate-related stressors, including both flooding and droughts of different types. The study’s results will also build on existing findings and survey results, such as the Public Health England [19], Royal Agricultural Benevolent Institution [15], Farm Safety Foundation (Yellow Wellies) [35], and NFU [27] studies, by providing a deeper understanding of how these events affect mental health in the United Kingdom.

### Strengths and Limitations

The strength of this study is the novel approach adopted to focus on the mental health of farmers and farmworkers related to flooding or drought together. We also use a mixed methods approach, which adds to this strength. The methodology does not include clinical or medical data from participants as this is not required for the intended outcomes.

A key limitation of this study is the reliance on self-reported data, which may introduce bias. Additionally, the survey is being distributed through intermediary organizations, which may result in a sample that is not fully representative of the wider farming population. However, this is intentional as intermediary organizations (in the agricultural and water sectors in this case) are likely more trusted by the community of interest compared to governmental departments in England. This may reduce bias for certain response topics. This study’s focus on farmers and farmworkers already affected by flooding and drought events may also restrict the applicability of the findings to wider agricultural or general populations or those who may be affected in the future.

### Conclusions

Findings from this study will contribute to the limited evidence and add new perspectives on mental health impacts to farmers and farmworkers in England from flooding and drought, as well as the coping strategies used. Findings from the mixed methods approach using an online survey and semistructured interviews will inform the development of targeted mental health interventions for farmers and farmworkers in England, helping build resilience for future flooding and drought events. The findings will also contribute to the growing body of research on the mental health impacts of climate change and support the creation of policies that address the unique needs of the agricultural community.

## Data Availability

Data sharing is not applicable to this paper as no datasets were generated or analyzed during this study.

## Appendix

Multimedia Appendix 1 Questionnaire.

Multimedia Appendix 2 Interview themes and topics.

Multimedia Appendix 3 Mental health support information.

## Abbreviations

NFU: National Farmers’ Union
WEMWBS: Warwick-Edinburgh Mental Wellbeing Scale
