# Supplementary material for: The Mental Health of Farmers and Farmworkers Impacted by Flooding and Drought: Protocol for a Mixed Methods Study
Source: JMIR Res Protoc. 2025 Dec 25;14:e73827. doi: 10.2196/73827 (PMC12784139; doi:10.2196/73827)
Supplement: Multimedia Appendix 1 [file resprot_v14i1e73827_app1.docx]

**Supplementary Material 1.**

**Stage 1 Questionnaire**

*Note. These are questions only. A consent form and information sheet are used before the questionnaire, but as this information largely reflects that in the study protocol, they have been omitted here for clarity. The below questions also omit question branching and inter-sectional information used in the Microsoft Forms version of the survey.*

Section 1: Demographic and Farm Information

1. What is your age?

- Under 18
- 18-24
- 25-34
- 35-44
- 45-54
- 55-64
- 65 and over

1. What is your gender?

- Man
- Woman
- Non-binary
- My gender is not listed
- Prefer not to say

1. Region:

*Note that the following regions refer to the 9 statistical regions in England (see map here:* [*https://www.gov.uk/government/statistics/country-and-regional-analysis-2021/country-and-regional-analysis-guidance#nuts-map-and-regions*](https://www.gov.uk/government/statistics/country-and-regional-analysis-2021/country-and-regional-analysis-guidance#nuts-map-and-regions)*)*

- East
- London
- East Midlands
- West Midlands
- North East
- North West
- South East
- South West
- Yorkshire and the Humber
- I do not live in England

1. Role in Agriculture:

- Farmer (Owner/Operator)
- Farm Worker (Employee)
- Tenant Farmer
- Contractor
- Other (please specify)

1. Type of Farming (select all that apply):

- Cereals
- Horticulture & Potatoes
- Specialist pigs
- Specialist poultry
- Dairy
- Upland Grazing Livestock
- Lowland Grazing Livestock
- Other (please specify)

1. How long have you been involved with farming in the area?

- 40 years or more
- 20 to 39 years
- 10 to 19 years
- 5 to 9 years
- Less than 5 years

1. Number of Farm Workers on Farm

*Note that non-rounded figures are to account for part-time or spare-time workers*

- Less than 1
- 1 to 1.99
- 2 to 2.99
- 3 to 4.99
- 5 or more

1. Does your farm use artificial irrigation?

- Yes
- No

1. If yes, what is the source of water for irrigation? (select all that apply)

- River or stream - direct
- Reservoir - fed from river or stream
- Reservoir - rainfall
- Mains water supply - water company
- Other (please specify)
- Prefer not to say

Section 2: Experience with Floods and Droughts

1. Have you experienced flooding on the farm in the past 5 years?

*Note that you should consider floods from weather-related events only, including river, coastal or surface water / overland flooding. Do not include floods from leaks or general infrastructure damage not related to weather events.*

1. Yes
2. No

1. What type of flooding have you experienced in the past 5 years?
2. Flooding from rivers or streams
3. Flooding from the coast or estuaries
4. Flooding from surface water or overland flow
5. Other – please specify
6. I don’t know

1. How many times and when did the flood/s occur?

For each flood event please provide the start date of the flooding and the number of days the floods impacted the farm land and/or property.

*If unsure, please provide an approximate date and duration where possible.*

1. Were you given any advance warning of flooding in your area by any source (radio, online, neighbours, Environment Agency)?
2. Yes
3. No
4. Unsure

1. How would you rate the severity of the flooding you experienced?
2. Not severe *(no impacts evident, all on-farm activities possible)*
3. Mildly severe (minor impacts, most on-farm activities possible)
4. Moderately severe (some impacts, some on-farm activities possible)
5. Very severe (major impacts, minimal on-farm activities possible)
6. Extremely severe *(extensive impacts/destruction, no on-farm activities possible)*

1. Did the flooding affect any assets or livestock on your property? (select all that apply)
2. Yes – Farmhouse (water entered ‘liveable rooms')
3. Yes – Other farm buildings
4. Yes – Land/fields/crops
5. Yes – Other assets (e.g., roads, tracks, machinery)
6. Yes – Livestock
7. No
8. Unsure

1. How long did the flood water directly affect these assets or livestock?

*(if you do not remember how long, please write your best guess (e.g., week or month).*  *Write N/A if it does not apply.*

1. Do you feel that flooding is becoming more of an issue for your farm?
2. Yes
3. No – It has stayed about the same
4. No – It has reduced

1. Have you experienced drought on the farm in the past 5 years?
2. Yes
3. No

1. How many times and when did the drought/s occur?

For each drought event please provide the start date of the event and the number of days the droughts impacted the farm land and/or property. *If unsure, please provide an approximate date and duration where possible.*

1. Were you given any advance warning of drought in your area by any source (radio, online, neighbours, Environment Agency)?
2. Yes
3. No
4. Unsure

1. How would you rate the severity of the drought you experienced?
2. Not severe *(no impacts evident, all on-farm activities possible)*
3. Mildly severe (minor impacts, most on-farm activities possible)
4. Moderately severe (some impacts, some on-farm activities possible)
5. Very severe (major impacts, minimal on-farm activities possible)
6. Extremely severe *(extensive impacts/destruction, no on-farm activities possible)*

1. Did the drought affect any assets or livestock on your property? (select all that apply)
2. Yes – Land/fields/crops
3. Yes – Livestock
4. No
5. Unsure

1. How long did the drought directly affect the farm?

*(if you do not remember how long, please write your best guess (e.g., week or month).*  *Write N/A if it does not apply.*

1. Do you feel that drought is becoming more of an issue for your farm?
2. Yes
3. No - It has stayed about the same
4. No – It has reduced

1. Have regulations limited the quantity of water required for irrigation?
2. Yes
3. No
4. Not applicable

1. Have you received (or are you due to receive) any financial support following floods or droughts in the past 5 years?
2. Yes
3. No
4. Unsure
5. If yes, what was the source of the funding and approximate value you received?

Section 3: Mental Health Impacts & Coping Strategies

1. How often do you feel stressed due to flooding or drought?
2. Never
3. Rarely
4. Sometimes
5. Often
6. Always

1. Have you experienced any of the following due to flooding or drought? (select all that apply)
2. Anxiety
3. Depression
4. Feelings of hopelessness
5. Social Isolation or Loneliness
6. PTSD (Post-traumatic stress disorder)
7. Insomnia
8. Fatigue
9. Irritability or Anger
10. Anger
11. Difficulty concentrating
12. Other (please specify)

1. What had negative impacts on your mental health following flooding or drought? (select all that apply)
2. Time away from home, family or friends.
3. Consequences for family, friends or community.
4. Insurance issues
5. Other financial difficulties
6. Damage or disruption to farm buildings
7. Damage or disruption to land / field / crops / livestock
8. Damage or disruption to other assets on the farm (e.g., roads, tracks, machinery)
9. Damage or disruption outside the farm (e.g., water supplies, electricity, storage facilities, transport, supply chains)
10. Issues with landowner or landlord/agent.
11. Regulatory conditions or government policy
12. Supply contract conditions
13. Knock-on impacts for the future (e.g., feed supply, yields)
14. Other (please specify)

1. What had positive impacts on your mental health following flooding or drought? (select all that apply)
2. Time with friends and family
3. Response and recovery activities with community
4. Insurance support
5. Support or funding from government or local authorities
6. Support or funding from farming charities or industry bodies
7. Support from landowner or landlord/agent
8. Resilience-building activities on the farm for future events
9. Resilience-building activities outside the farm for future events
10. Flexibility with regulatory conditions or government policy
11. Flexibility with supply contract conditions
12. Other (please specify)

1. Have you sought or will you seek professional help for mental health issues related to flooding and drought challenges?
2. Yes
3. No
4. Unsure

1. If you sought help, what type of support did you seek? (select all that apply)
2. Family or friends
3. Financial
4. GP/Doctor
5. Hospital
6. Therapist/Counselor
7. Support group or charity
8. NHS 111 or Helpline
9. Other (please specify)

1. Do you have any other coping mechanisms for mental health issues?
2. Yes – *please give details if willing to share*
3. No

1. Do you feel you have adequate support to manage your mental health?
2. Yes
3. No
4. Unsure

1. What strategies do you use to cope with stress or mental health issues related to flooding and drought? (Select all that apply)
2. Talking to family/friends
3. Physical exercise
4. Meditation/Relaxation techniques
5. Professional support or helplines
6. Community, charity or social support groups
7. Prescribed medication
8. Alcohol or non-prescribed drugs
9. Other (please specify)

1. What additional support would be most helpful to you in managing the mental health impacts from flooding and drought? (Select all that apply)
2. Improved access to mental health services
3. Financial support
4. Changes to insurance
5. Training in stress management
6. Community support networks
7. Government policy changes
8. Other (please specify)

1. Who should have overall responsibility for providing this additional support?
2. Farm owners or workers
3. NHS or GPs
4. Farming organisations
5. Local or regional government
6. National government
7. Charities or third sector
8. Private sector
9. Other (please specify)

Section 4: Warwick-Edinburgh Mental Wellbeing Scale *(optional)*

There are 14 multiple choice questions in this final section of the questionnaire that were developed by an expert panel over 15 years ago. They comprise only of positively worded questions relating to different aspects of mental health and wellbeing.

There are five choices for the answer to each question as you will see. Please only tick one box for each.

Please use the answers to these questions to describe your experiences over the past two weeks only. This two-week timeframe is to align with the standard requirements of this scale.

1. I’ve been feeling optimistic about the future.

- None of the time
- Rarely
- Some of the time
- Often
- All of the time

1. I’ve been feeling useful.

- None of the time
- Rarely
- Some of the time
- Often
- All of the time

1. I’ve been feeling relaxed.

- None of the time
- Rarely
- Some of the time
- Often
- All of the time

1. I’ve been feeling interested in other people.

- None of the time
- Rarely
- Some of the time
- Often
- All of the time

1. I’ve had energy to spare.

- None of the time
- Rarely
- Some of the time
- Often
- All of the time

1. I’ve been dealing with problems well.

- None of the time
- Rarely
- Some of the time
- Often
- All of the time

1. I’ve been thinking clearly.

- None of the time
- Rarely
- Some of the time
- Often
- All of the time

1. I’ve been feeling good about myself.

- None of the time
- Rarely
- Some of the time
- Often
- All of the time

1. I’ve been feeling close to other people.

- None of the time
- Rarely
- Some of the time
- Often
- All of the time

1. I’ve been feeling confident.

- None of the time
- Rarely
- Some of the time
- Often
- All of the time

1. I’ve been able to make up my own mind about things.

- None of the time
- Rarely
- Some of the time
- Often
- All of the time

1. I’ve been feeling loved.

- None of the time
- Rarely
- Some of the time
- Often
- All of the time

1. I’ve been interested in new things.

- None of the time
- Rarely
- Some of the time
- Often
- All of the time

1. I’ve been feeling cheerful.

- None of the time
- Rarely
- Some of the time
- Often
- All of the time

Thank you for completing those questions.

- Reflecting on the questions, are there any that you would have answered differently closer to the time of the flood and/or droughts that you have experienced on the farm?
- Yes
- No

- If yes, please indicate which ones and explain why *(open feedback answer)*

Section 5: Open Feedback

Please provide any additional comments or suggestions on how to better support farmers and farm workers dealing with mental health impacts of flooding and / or drought events:

Section 6: Further Information and Research

I would like to receive a summary of key findings from the questionnaires completed (sent within 2 months)

1. Yes
2. No

I wish to support this study further through a potential follow-up interview and agree to providing my contact details and being contacted within 12 months of this questionnaire to arrange a suitable time.

1. Yes
2. No

*Note that follow-up interviews are not guaranteed as they will depend on time and resources within the research team.*

Full Name:

Email Address:

Phone number (Landline):

Phone number (Mobile):

SUBMISSION

End: Thank you for taking the time to complete this questionnaire. Your feedback is invaluable in helping us understand the mental health impacts of flooding and drought events and to develop better support systems for those affected.

If you feel that you need support following this survey, we advise to contact your GP. There are also resources available to help:

- NHS Mental Health Hotline: For immediate mental health support, call 111 and select the mental health option or visit [www.nhs.uk/mental-health](https://www.nhs.uk/mental-health).
- Samaritans: Available 24/7 if you need someone to talk to. Call 116 123 or visit [www.samaritans.org](http://www.samaritans.org/).
- Farming Community Network (FCN): A charity providing pastoral and practical support for farmers. Call 03000 111 999 or visit [www.fcn.org.uk](https://www.fcn.org.uk/).
- Mind: For mental health support and information. Call 0300 102 1234 or visit [www.mind.org.uk](http://www.mind.org.uk/).
- Shout: This is a free, confidential 24/7 text service where you can speak to a trained volunteer who can help with mental health issues such as anxiety, depression, suicidal thoughts, and more. Text “SHOUT” to 85258.
- Royal Agricultural Benevolent Institution (RABI): Providing financial, practical, and emotional support to farmers and their families. Call 0800 188 4444 or visit [www.rabi.org.uk](http://www.rabi.org.uk/).
- Yellow Wellies: The Farm Safety Foundation: Offering mental health resources and support for the farming community. Visit [www.yellowwellies.org](http://www.yellowwellies.org/).
- National Federation of Young Farmers’ Clubs (NFYFC): Supporting young farmers with a range of resources, including mental health. Visit [www.nfyfc.org.uk](http://www.nfyfc.org.uk/) for more information.
- Hub of Hope: The UK’s leading mental health support database, bringing together a range of support and services including those local to your address. Visit [www.hubofhope.co.uk](http://www.hubofhope.co.uk/).

Please reach out if you need assistance. Your well-being is important, and support is available.
