# Supplementary material for: The Mental Health of Farmers and Farmworkers Impacted by Flooding and Drought: Protocol for a Mixed Methods Study
Source: JMIR Res Protoc. 2025 Dec 25;14:e73827. doi: 10.2196/73827 (PMC12784139; doi:10.2196/73827)
Supplement: Multimedia Appendix 2 [file resprot_v14i1e73827_app2.docx]

**Supplementary Material 2.**

**Stage 2 Semi-Structured Interview Themes and Topics**

*Note. These are proposed topics and themes only and are subject to change after stage 1 survey responses and further stakeholder discussions. Question prompting will also adapt as the interviews progress given the semi-structured nature allowing for 2-way conversations. Planned introductory and general information has been omitted for clarity.*

**Demographic Information**

Confirmation of information collected during the questionnaire as some time may have passed and details may have changed.

- Discussion on farm type, farm size (number of employees and hectares)
- Discussion on the flood and/or drought events mentioned in questionnaire. Clarify if multiple events and their spatial and temporal characteristics. Any further events or concerns since the time of the questionnaire?
- Prompt for level of stress and worry from following sources:
- Government policy
- Regulations including water use
- Social isolation
- Succession planning
- Brexit
- Cost of products/labour
- Community sustainability
- Supermarket supplier agreements.

**Personal Impacts - General**

- Initial reactions and emotions.
- Can you describe your initial feelings when the flooding / drought occurred?
- I see that you listed the following mental health impacts in the questionnaire… Would you like to expand on these anymore?

- Coping Mechanisms
- I see that you listed the following coping strategies in the questionnaire… Can you explain a bit more about the strategies or activities you have you found helpful and not so helpful in managing mental health impacts to flooding and/or drought?
- And you felt you did / did not have adequate support to manage your mental health.
- What do you think are the key challenges here? Do you think these challenges apply to others in your community?

Or

- That’s promising to hear – what do you think helped with the provision of support? Do you think it applies to others in your community?

**Warwick-Edinburgh Mental Wellbeing Scale**

There are 14 questions in this section that were developed by an expert panel over 15 years ago. They comprise only of positively worded questions relating to different aspects of mental health and wellbeing.

We will give you a sheet and questions for you to complete. Please take as much time as you need to complete these now. Alternatively, we can read the questions out if you prefer?

There are five choices for the answer to each question as you will see. Please only tick one box for each.

As a reminder, please use the answers to these questions to describe your experiences over the past two weeks only.

**Warwick-Edinburgh Mental Wellbeing Scale**

**Question Sheet**

*Please use the answers to these questions to describe your experience* ***over the past two weeks only.*** *As with all interview questions, these are optional.*

1. I’ve been feeling optimistic about the future.

- None of the time
- Rarely
- Some of the time
- Often
- All of the time

1. I’ve been feeling useful.

- None of the time
- Rarely
- Some of the time
- Often
- All of the time

1. I’ve been feeling relaxed.

- None of the time
- Rarely
- Some of the time
- Often
- All of the time

1. I’ve been feeling interested in other people.

- None of the time
- Rarely
- Some of the time
- Often
- All of the time

1. I’ve had energy to spare.

- None of the time
- Rarely
- Some of the time
- Often
- All of the time

1. I’ve been dealing with problems well.

- None of the time
- Rarely
- Some of the time
- Often
- All of the time

1. I’ve been thinking clearly.

- None of the time
- Rarely
- Some of the time
- Often
- All of the time

1. I’ve been feeling good about myself.

- None of the time
- Rarely
- Some of the time
- Often
- All of the time

1. I’ve been feeling close to other people.

- None of the time
- Rarely
- Some of the time
- Often
- All of the time

1. I’ve been feeling confident.

- None of the time
- Rarely
- Some of the time
- Often
- All of the time

1. I’ve been able to make up my own mind about things.

- None of the time
- Rarely
- Some of the time
- Often
- All of the time

1. I’ve been feeling loved.

- None of the time
- Rarely
- Some of the time
- Often
- All of the time

1. I’ve been interested in new things.

- None of the time
- Rarely
- Some of the time
- Often
- All of the time

1. I’ve been feeling cheerful.

- None of the time
- Rarely
- Some of the time
- Often
- All of the time

Thank you for completing those questions.

- Reflecting on the questions, are there any that you would have answered differently closer to the time of the flood and/or droughts that you have experienced on the farm?

Thank you. Moving on to the next part of the overall interview now, it would be great if you are happy to discuss some of the impacts experienced in more detail.

**Farm Impact**

- Operational Changes
- How have flooding and drought conditions impacted your farming operations?
- What changes have you had to make to adapt to these conditions?

- Economic Impact
- Can you discuss the financial effects of these events on your farm?
- How have these financial strains affected your mental health?

- Future Planning
- How do you plan for future flooding or drought events?
- Has the threat of these events changed your long-term plans for your farm?

**Community Impact**

- Community Support
- How has your local community responded to flooding/drought events?
- Have community networks provided support, and if so, how?

- Social Relationships
- Have the floods and/or droughts affected your relationships with neighbours, friends, or family?
- Is there a sense of increased solidarity or tension within your community?

- Collective Mental Health
- What have you observed about the mental health of other farmers and farmworkers in your community?
- Are there any community-wide initiatives to address mental health concerns related to these events?

**Resources and Assistance**

- Access to Resources
- What resources (financial, technical, or psychological) have you accessed to help cope with flooding/drought?
- Are there resources you wish were more readily available?

- Effectiveness of Assistance
- How effective have government programmes or NGO interventions been in providing support?
- What improvements do you think could be made to these support systems?

- Barriers to Support
- Have you encountered any barriers to accessing mental health or other support services?
- How could these barriers be reduced or eliminated?

**Policy and Advocacy**

- Policy Awareness
- Are you aware of any policies or programmes aimed at supporting farmers affected by flooding and drought?
- How do you feel about the adequacy of these policies?

- Advocacy and Change
- What changes would you advocate for to better support farmers facing these challenges?  *(prompt to consider different levels including wider agricultural community)*
- How do you think policymakers and the wider public could better understand and address the mental health impacts on farmers and farmworkers?

**Future Outlook**

- Resilience
- What resilience measures are you taking to protect your farm and business activities against future floods and droughts?
- How optimistic or pessimistic are you about the future in terms of both farming and personal mental health?

- Is there anything else you would like to discuss?  *(Could prompt for general hopes and concerns moving forward)*

Thank you very much for your time. If you do think of something else we ought to be aware of, please do let us know either by phone or email.

END
