# Supplementary material for: The Mental Health of Farmers and Farmworkers Impacted by Flooding and Drought: Protocol for a Mixed Methods Study
Source: JMIR Res Protoc. 2025 Dec 25;14:e73827. doi: 10.2196/73827 (PMC12784139; doi:10.2196/73827)
Supplement: Multimedia Appendix 3 [file resprot_v14i1e73827_app3.docx]

**Supplementary Material 3.**

**Mental Health Support Information**

**(provided before and after the survey and interviews)**

If you feel that you need support following this survey, we advise to contact your GP. There are also resources available to help: ·

- **NHS Mental Health Hotline**: For immediate mental health support, call **111** and select the mental health option or visit www.nhs.uk/mental-health.
- **Samaritans**: Available 24/7 if you need someone to talk to. Call **116 123** or visit www.samaritans.org.
- **Farming Community Network (FCN)**: A charity providing pastoral and practical support for farmers. Call **03000 111 999** or visit www.fcn.org.uk.
- **Mind**: For mental health support and information. Call **0300 102 1234** or visit www.mind.org.uk.
- **Shout:**This is a free, confidential 24/7 text service where you can speak to a trained volunteer who can help with mental health issues such as anxiety, depression, suicidal thoughts, and more.**Text “SHOUT” to 85258.**
- **Royal Agricultural Benevolent Institution (RABI)**: Providing financial, practical, and emotional support to farmers and their families. Call **0800 188 4444** or visit www.rabi.org.uk.
- **Yellow Wellies**: The Farm Safety Foundation: Offering mental health resources and support for the farming community. Visit www.yellowwellies.org.
- **National Federation of Young Farmers’ Clubs (NFYFC)**: Supporting young farmers with a range of resources, including mental health. Visit www.nfyfc.org.uk for more information.
- **Hub of Hope**: The UK’s leading mental health support database, bringing together a range of support and services including those local to your address. Visit www.hubofhope.co.uk

Please reach out if you need assistance. Your well-being is important and support is available.
